# Early combined parenteral and enteral nutrition support for patients over 80 years old with non-operative intestinal obstruction

**DOI:** 10.3389/fnut.2026.1762338

**Published:** 2026-04-28

**Authors:** Zhida Chen, Pengfei Zhang, Yi Liu, Hongqing Xi

**Affiliations:** 1 Department of General Surgery, First Medical Center of Chinese PLA General Hospital, Beijing, China; 2 Department of Oncology, Chinese PLA General Hospital, Beijing, China; 3 Chinese PLA Medical School, Beijing, China

**Keywords:** advanced age, enteral nutrition, intestinal obstruction, non-operative treatment, nutrition support

## Abstract

**Objective:**

We aimed to evaluate the security and application value of early combined parenteral and enteral nutrition support for non-operative intestinal obstruction in patients aged over 80 years old.

**Methods:**

Data on 191 older patients who were receiving combined parenteral and enteral nutrition support from August 2008 to July 2019 were retrospectively recorded and examined. According to the starting time of enteral nutrition support, we divided the patients into two groups: early parenteral nutrition combined with enteral nutrition (ECPEN, *n* = 85) and delayed parenteral nutrition combined with enteral nutrition (DCPEN, *n* = 106). According to all groups’ history data, NRS2002 and mini nutritional assessment (MNA) were employed for nutritional risk screening and malnutrition classification. After propensity score matching (PSM), 79 sets of data were included in further statistics. The comparison results included intestinal obstruction treatment, nutritional support, infection index, biochemical index and complications.

**Results:**

A total of 79 pairs were selected after 1:1 propensity score matching based on gender, age, BMI, nutritional score, history of abdominal surgery, complications, and performance status. The baseline levels of patients with ECPEN and DCPEN were basically balanced. There were no significant differences in the inhibitor use time or the time of first flatulation and defecation between the two groups, but the duration of total parenteral nutrition (TPN) in the DCPEN group was longer, while the time of combined application of PN and EN was shorter. Enteral nutrition (EN) was applied earlier in the ECPEN group than in the DCPEN group, and EN gradually increased to 60% of the target energy requirement. In the ECPEN group, the average hospitalization days were less and the medical expenses were low. After seven days of non-surgical treatment, the white blood cells, lymphocytes, IL-6, PCT, ALB, blood sodium, blood potassium, and blood phosphorus of the two groups all improved, with significant differences compared with before the treatment. Blood glucose content in the ECPEN group gradually declined to an acceptable range from a high and fluctuated level, with a smaller standard deviation and better stability compared to the DCPEN group. The ALT and AST values in the ECPEN group returned to a normal range, but the liver function indexes in the DCPEN group were still higher than the upper limit of the normal range. Within 30 and 180 days after operation, there were fewer class I-IV complications in the ECPEN group than in the DCPEN group, and the incidence of class II complications was the most significantly different (10 vs. 22 and 15 vs. 25, respectively). After 30, 90, and 180 days of treatment, the readmission rate of patients in the ECPEN group was also lower than that in the DCPEN group, with statistical significance.

**Conclusion:**

Old age is not a contraindication to early enteral nutrition for incomplete intestinal obstruction. Estimating the time to apply EN accurately and arranging total amount of intravenous fluid based on the patient’s organic function can benefit elderly patients and also be an important nutritional support for incomplete ileus through non-operative treatment.

## 1 Introduction

Life expectancy has risen this century, leading to a growing population of people over 80. Therefore, the rate of emergency admissions for patients over 80 years old continues to rise (1–5). Many elderly people suffer from gastrointestinal dysfunction and intestinal obstruction, including malignant and incomplete intestinal obstruction. For incomplete intestinal obstruction, conventional treatment, including nutritional support, antibiotics, and anti-secretory drugs, is effective without surgical intervention. Age and comorbidity affect the results, and their synergistic effect is obvious in elderly patients (6–8). The latest data show that 83% of acute geriatric patients are diagnosed as malnourished, and targeted nutritional intervention leads to a significant reduction in hospitalization time and representativeness. The cardiopulmonary function of the elderly is complicated, and the flow of fluid replacement is limited (9–15). In addition, short-term fasting and water-deficient diet therapy may lead to worsening malnutrition (16–19). Therefore, early enteral nutrition is closely related to survival and quality of life (QOL). Clinicians should determine appropriate and personalized treatment programs for elderly patients (20, 21).

Malnutrition is common in elderly patients suffering from intestinal obstruction and, if parenteral nutrition is preferred, early combined enteral nutrition is prescribed (22). The data on nutrition support outcomes for non-operative intestinal obstruction in patients aged over 80 years old are lacking, and accurate data on treatment outcomes are essential to guide therapeutic decision-making.

In this study, we aim to compare the infection, biochemical results, and related complications of early or delayed combined parenteral and enteral nutritional support therapy in patients over 80 years old.

### 1.1 The acquisition of medical records and materials

#### 1.1.1 Patient information

The current study is a retrospective analysis of from the database from PRIDE® Electronic Health Record System (EHR-S). From August 2008 to July 2019, 191 patients with non-operative intestinal obstruction over 80 years old who started and completed parenteral and enteral nutrition support in the general surgery department of the First Medical Center of the People’s Liberation Army (PLA) General Hospital were recruited. These patients were studied and analyzed after strict screening. Medical data and nutritional details were comprehensively entered into this database by an experienced clinical data manager.

#### 1.1.2 Inclusion and exclusion criteria

##### 1.1.2.1 Inclusion criteria

(1) patients over 80 years old; (2) acute onset, no exhaust and defecation, with typical clinical symptoms such as abdominal pain, vomiting, and abdominal distension; (3) abdominal X-ray plain film, CT scan, or other auxiliary examinations that meet the diagnostic criteria of acute incomplete intestinal obstruction; (4) patient’s vital signs are stable and can be treated conservatively.

##### 1.1.2.2 Exclusion criteria

(1) dietetic records were unavailable; (2) parenteral nutrition resistance accompanied by abnormal liver function; (3) strangulated intestinal obstruction caused by intussusception, volvulus, or inflammatory stenosis intestine; (4) mechanical intestinal obstruction caused by fecalith, malignant carcinoma, or foreign matter in the intestine; (5) necrotizing intestinal obstruction caused by acute mesenteric ischemia; (6) end-stage tumor patients; or (7) conversion to surgical intervention when conservative treatment failed.

#### 1.1.3 Nutrition intervention measures

Each patient received an individual nutrition assessment by a registered dietitian and received corresponding treatment. After adjusting according to the stress and activity level, the personal energy demand was calculated.

Hospitalized elderly patients with non-operative intestinal obstruction received parenteral nutrition, glucose and saline infusions, antibiotics, and antisecretory agents. The primary difference between the two groups was the timing of enteral nutrition initiation. Early parenteral nutrition combined with enteral nutrition (ECPEN) involved a small amount of enteral nutrition, via oral or tube feeding, immediately after the first defecation, flatulence, abdominal distension, or normal bowel sounds that showed signs of partial relief. According to the patient’s tolerance, the amount of enteral nutrition increased, while the input of parenteral nutrition decreased synchronously. Delayed combined parenteral and enteral nutrition (DCPEN) involved a small amount of enteral nutrition several days after the above clinical symptoms appeared. During hospitalization, patients were given TPN most of the time. Specifically, standard whole-protein Oral Nutritional Supplements (ONS) preparations were utilized. For patients receiving enteral tube feeding, the nutrient solution was administered via an enteral feeding pump with a cautious initial infusion rate of 10–20 mL/h. For patients who tolerated oral feeding, a small initial dosage of 300–500 mL/day was provided, diluted to 1/3 or 1/2 of the standard concentration if necessary. The progression of the infusion rate or oral intake volume was gradually increased based on dynamic monitoring of the patient’s gastrointestinal tolerance (e.g., absence of severe abdominal distension, vomiting, or diarrhea) until the target energy requirement was reached.

#### 1.1.4 Primary and secondary indexes

The primary indexes included two aspects: 1. Nutritional support: the start time of enteral nutrition feeding, the number of days of enteral nutrition treatment to reach 60% of the target energy, the total amount of parenteral nutrition, and the configuration components; and 2. Objective physical indicators: control of inflammatory indicators (white blood cells, lymphocytes, CRP, IL-6, and PCT), liver function (albumin, TC, triglyceride, LDL, HDL, ALT, and AST), serum electrolyte ion level, blood sugar level, and pH value.

Secondary indexes included observation, total length of hospitalization, hospitalization expenses, short-term complications, and readmission rate.

#### 1.1.5 Nutrition assessment tools

NRS2002 and MNA were used to classify nutritional risk screening and malnutrition.

##### 1.1.5.1 NRS2002

Three aspects from the NRS2002 scale were used to evaluate older patients: nutritional status on admission, recent body quality change, primary disease severity, and age ≥ 70 years old. The total score is the sum of the points for each item (0–7 points). The higher the score, the worse the nutritional status. A score of 3 or higher would be categorized as a nutritional risk.

##### 1.1.5.2 MNA

By consulting the MNA questionnaire, the elderly patients were evaluated from four aspects, namely anthropometry (body mass index, calf circumference, upper arm circumference, and decreased body mass over the past three months), overall evaluation (psychology, life style, medical care, and health and disease), dietary evaluation (daily food intake and feeding behavior and loss of appetite), and subjective evaluation (evaluation of their nutrition and health by themselves and others). The risk of malnutrition was divided into three grades according to the score: those with MNA above 24.0 were well nourished; those with an MNA between 17 and 23.5 were at risk of malnourishment; and an MNA below 17 was malnourished.

#### 1.1.6 Post-discharge follow-up

According to the 30-day, 90-day and 180-day discharge time nodes, the hospitalized patients were followed up by telephone and outpatient service. During the follow-up process, doctors were willing to give family nutritional support and record complications after discharge or readmission. Short-term complications after discharge were recorded according to the Calvin-Dindo grading method (23).

(I) Any deviation from the normal recovery process but no medication, surgical intervention, endoscopy, or interventional therapy is required; (II) Drug treatment including blood transfusion, antibiotics, and total parenteral nutrition; (III) Surgery, endoscopy, or interventional therapy; and (IV) Life-threatening, processed in the intensive care unit.

#### 1.1.7 Statistical methods and propensity score matching

Statistical analysis and propensity score matching were performed using SPSS 22.0, utilizing a 1:1 nearest neighbor matching method. Quantitative data (Mean ± SD) t-test was used to compare between different groups. A comparison between the disorderly classification data set was performed using the χ^2^ test or Fisher exact probability test, while an orderly classification comparison between data sets was performed using Wilcoxon rank sum test. *p* < 0.05 was statistically significant.

## 2 Results

### 2.1 Patient information and baseline correction

We carried out 1:1 proximity matching regarding the patient’s gender, age, BMI, nutrition score, medical history of abdominal operation, complications, and performance. After that, 79 pairs of data were selected. The baseline levels of the above seven factors were equilibrated between ECPEN and DCPEN patients, with no significant difference but good comparability (*p* > 0.05).

### 2.2 Treatment and nutritional support for intestinal obstruction

Both groups of elderly patients with intestinal obstruction received routine treatments, including enzyme inhibition, acid suppression, and anti-infective therapy. After non-surgical treatment, there were no significant differences in inhibitor use time, time until first flatulence, and the time until using antibiotics to defecate. From the point of view of PN support, TPN support time in the DCPEN group was longer than that in the ECPEN group, while the cooperation time between PN and EN was shorter. From the point of view of EN supplement, the ECPEN group required EN earlier than the DCPEN group, showing a slow increase trend, reaching 60% of the target requirement, while the DCPEN group required EN in a rapid way after the long-term TPN reached more than 60%.

In addition, ECPEN was superior to DCPEN in health economics and health care, as the average hospitalization time was shorter and the medical expenses were lower.

### 2.3 Comparison of hematological indexes before and after treatment

Seven days post-treatment, a comparison of hematological parameters revealed that markers such as white blood cell count, lymphocytes, IL-6, PCT, albumin, sodium, potassium, and phosphate had all significantly improved compared to baseline. CRP and PH values in both groups also returned to the normal range, but there was no significant change to before the treatment. Blood glucose content in the ECPEN group gradually declined to an acceptable range from a high and fluctuated level, with a smaller standard deviation and better stability compared to the DCPEN group. The ALT and AST values (liver function index) in the ECPEN group returned to a normal range, but they were still higher than the upper limit of the normal range in the DCPEN group.

### 2.4 Complications and short-term follow-up

We classified patients’ short-term complications and readmissions using the Clavien-Dindo classification system. As far as short-term complications are concerned, within 30 and 180 days after treatment, the class I I-IV complications in the ECPEN group were less than those in the DCPEN group, which was statistically significant. The differences of grade II complications were the most significant, namely 10 vs. 22 and 15 vs. 25, respectively, in these two time periods. Within 30 days, 90 days, and 180 days, the readmission rate in the ECPEN group was lower than that in DCPEN group, with statistical significance.

## 3 Discussion

Incomplete intestinal obstruction is a common condition among elderly patients and is classified into functional and non-functional types. The main symptoms in the functional categories are the weakening of gastrointestinal function and the decrease of emptying ability and intestinal activity. The non-functional category is mainly cases of mild and moderate intestinal obstruction adhesion. Research shows that 75% of small intestinal obstruction is related to postoperative adhesion (24). In addition to operation-related adhesions, non-functional intestinal obstruction can also be secondary to abdominal trauma, infectious or non-infectious abdominal pelvic inflammatory disease, abdominal cavities, and foreign bodies. The above causes of incomplete intestinal obstruction often do not need surgery but can be alleviated through gastrointestinal decompression, inhibiting gastric acid, inhibiting pancreatic enzyme secretion, nutritional support, and auxiliary antibiotics.

### 3.1 Nutritional risk screening and assessment in octogenarians

Nutritional risk refers to existing or potential risks related to nutritional factors that lead to adverse clinical results for patients. Malnutrition is mainly due to a lack of nutrient intake, malabsorption, or stress response, such as disease, trauma, and infection, in which nutrient consumption increases. In patients with acute incomplete intestinal obstruction, especially elderly patients, acute abnormal carbohydrate metabolism, increased protein conversion rate, lipolysis, decreased fat storage, decreased lean body mass, muscle and visceral protein consumption, changes in energy consumption, water-electrolyte disorders, and local factors such as digestion, absorption, and barriers can aggravate nutritional risks and malnutrition (25). However, nutritional risk and malnutrition, survival rates, mortality and complications, hospitalization time, and cost are closely related to the clinical results. Therefore, proper nutritional therapy for elderly patients with nutritional risk and malnutrition can improve their clinical prognosis (26, 27).

Using appropriate methods and tools to accurately identify malnutrition (or malnutrition risk) is necessary to guide nutrition diagnosis and treatment plans. At present, a variety of nutrition risk screening and assessment tools have been developed. However, the specific medical environment, effectiveness, and operability should be considered in the target evaluation when selecting tools.

Common nutrition risk screening tools include nutrition risk screening 2002 (NRS2002), malnutrition universal screening tools (MUST), the malnutrition screening tool (MST), and NUTRIC score. Nutritional assessment methods include patient-generated subjective global assessment (PG-SGA), subjective global assessment (SGA), mini nutritional assessment (MNA), and the geriatric nutritional risk index (GNRI) (28).

Based on the NRS2002 rating of 128 items of evidence-based medicine, using strong evidence-based medicine is simple and easy to use, and ESPEN recommends it as the first choice for inpatient malnutrition risk screening tool (29). It is advised not to rely on special clinical testing index with fast and straightforward characteristics. Therefore, MUST is recommended to be used in an outpatient or community health care environment. MST includes several testing aspects, such as loss of appetite and weight, and is mainly used for outpatient cancer screening (30). NUTRIC score is a specific nutrition risk screening tool for critically ill patients (31). Our primary object of study was inpatients. Therefore, we chose NRS 2002 as a nutrition risk screening tool because it has better applicability.

Regarding nutritional assessment, the MNA comprises 18 items and demonstrates high accuracy (32). Many evidence-based studies show that MNA can accurately evaluate the nutritional status of hospitalized elderly patients in relation to adverse clinical results (33–35). Therefore, in 2009, ESPEN recommended a comprehensive assessment method based on MNA assessment for elderly patients with malnutrition (36–38). PG-SGA is recommended by ASPEN as a noninvasive, rapid, and simple nutritional assessment method, which is mainly used as a specific nutritional assessment tool for cancer patients (39, 40). SGA operation is repeatable and straightforward but depends to a certain extent on evaluating subjective judgment-related indicators, so the accuracy of SGA results is poorly presented (41). Based on the comprehensive consideration of object accuracy and application, we chose MNA as a nutritional evaluation tool for elderly patients (Table 1).

**Table 1:** Patient information and baseline before PSM.

General situation		ECPEN group	DCPEN group	*p* value
Sex				0.016
	Male	47	59	
	Female	38	47	
Age				0.035
	80–89	64	73	
	≥90	21	33	
BMI		22.9 ± 2.3	23.8 ± 1.9	0.047
Nutrition score				
	NRS2002	3.7 ± 2.6	3.2 ± 2.9	0.031
	MNA-SF	18.9 ± 6.7	16.7 ± 7.6	0.015
History of abdominal operation				0.152
	Gastrointestinal	9	13	
	HBP	6	8	
	Urologic	2	1	
	Gynecological	6	10	
Co-morbidity				0.044
	Cardiac disease	57	68	
	Diabetes mellitus	33	41	
	Pulmonary disease	24	34	
	Hepatic disease	7	12	
	Kidney disease	10	9	
Locomotivity				0.024
	Ambulant	18	29	
	Immobilization	67	77	

HBP, hepato-bilio-pancreatic. Cardiac diseases include hypertension, coronary heart disease, and hyperlipidemia. Pulmonary diseases include lung carcinoma, chronic obstructive pulmonary disease/emphysema, pneumonia, and hydrothorax. Hepatic diseases include fatty liver, liver cirrhosis, hepatic carcinoma, and hepatic insufficiency. Kidney diseases include diabetic nephropathy, hypertensive nephropathy, chronic renal failure, chronic nephrotic syndrome, nephrotic syndrome, acute renal injury, and urinary tract infection. Immobilization includes the use of a wheelchair, stretcher, or transfer bed.

### 3.2 Clinical advantages of early combined nutrition

Elderly patients with malnutrition or at nutritional risk require nutritional support treatment. Patients who opt for enteral nutrition (EN) first should be given supplementary parenteral nutrition (SPN) if they cannot tolerate enteral nutrition, starve for more than 3 days, or simple enteral nutrition cannot reach 60% of the target demand within 7 days (25, 42). Compared with parenteral nutrition, enteral nutrition is more in line with natural physiology as it maintains the barrier function of the gastrointestinal tract, reduces liver damage, and simplifies the treatment of blood sugar problems. Different gastrointestinal function states give different enteral nutrition choices, such as oral nutritional supplements (ONS) or enteral tube feeding (ETF). Total parenteral nutrition (TPN) is the only nutrition option for patients who completely exclude the absorption function of the gastrointestinal tract (43). TPN is suitable for EN patients with absolute contraindications such as complete mechanical intestinal obstruction, uncontrollable peritonitis, ischemic bowel disease, severe shock, severe anastomotic leakage, or gastrointestinal failure. In addition, EN can still be used cautiously despite traditional contraindications to it, such as non-mechanical intestinal obstruction, open abdominal cavity, early intestinal fistula, gastrointestinal bleeding, intestinal wall edema, or using a pressor to maintain blood pressure stability. Many research results show that, compared with TPN, EN can reduce infection complications, shorten hospitalization time, and reduce medical expenses for elderly patients with sugar and electrolyte disorders, related liver disease, intestinal dysfunction, metabolic bone disease, and overfeeding syndrome. Therefore, we understand that age is not a contraindication to early use of enteral nutrition (44).

Elderly patients with incomplete intestinal obstruction in the early acute phase who were unable to tolerate oral intake, failed to supplement with ONS, or could not meet 60% of their nutritional requirements via tube feeding were immediately started on TPN. If the obstructive disease improves, and symptoms such as exhaustion, defecation, and other bowel sounds become more regular, we can adjust the nutritional support strategies by changing from TPN with EN as the main component to SPN, as well as attempting small ONS or ETF dosages and gradually increasing the amount of EN. When the total energy demand of EN reaches 60%, SPN should be lowered to supplement energy and liquid, and PN should be cut off to prevent overfeeding (Table 2).

**Table 2:** Patient information and baseline after PSM.

General situation		ECPEN group	DCPEN group	*P* value
Sex				0.375
	Male	42	44	
	Female	37	35	
Age				0.736
	80–89	61	59	
	≥90	18	20	
BMI		23.2 ± 2.2	23.5 ± 1.7	0.293
Nutrition score				
	NRS2002	3.6 ± 2.5	3.4 ± 2.8	0.177
	MNA-SF	19.1 ± 6.3	18.2 ± 7.4	0.132
History of abdominal operation				0.503
	Gastrointestinal	7	8	
	HBP	6	5	
	Urologic	2	1	
	Gynecological	5	6	
Co-morbidity				0.292
	Cardiac disease	51	49	
	Diabetes mellitus	30	32	
	Pulmonary disease	22	25	
	Hepatic disease	6	10	
	Kidney disease	9	7	
Locomotivity				0.279
	Ambulant	17	19	
	Immobilization	62	60	

Taking EN earlier can bring many benefits to patients, provide nutritional substrate, reduce the acute attack of hypercatabolic reactions and insulin resistance, reduce the release of inflammatory mediators, promote anabolism, prevent intestinal bacterial translocation, and maintain intestinal mucosal barrier and immune function (27, 45, 46). However, EN launch timing depends on the patient’s gastrointestinal function recovery situation and should be begun with caution after a comprehensive evaluation by a nutrition support team (NST). In principle, the patient’s tolerance should be gradually increased from a low concentration and dose. Early EN should be accompanied by the use of standard whole protein ONS preparations, including 1/3 to 1/2-low concentration if necessary, at small doses of 300–500 mL per day, and the starting speed of the tube-fed nutrient solution pump should be 10–20 mL/h. Dynamic monitoring of abdominal distension, vomiting, diarrhea, and other adverse reactions should be observed to provide timely symptomatic treatment (Table 3).

**Table 3:** Ileus treatment and nutrition support.

Treatment situation		ECPEN group	DCPEN group	*P* value
Antibiotics used		*n* = 65	*n* = 68	0.381
Antisecretory agent		*n* = 74	*n* = 72	0.245
First flatus (d)		2.8 ± 1.3	3.3 ± 1.7	0.193
First defecation (d)		3.4 ± 1.8	3.7 ± 2.2	0.111
PN infusion pathway				0.339
	PPN	*n* = 70	*n* = 67	
	Central venous	*n* = 9	*n* = 12	
EN infusion pathway				0.651
	ONS	*n* = 74	*n* = 71	
	Tube feeding	*n* = 5	*n* = 8	
Nutrition duration (d)				
	TPN	3.1 ± 1.1	5.7 ± 3.2	0.008
	PN + EN	3.5 ± 1.5	2.4 ± 1.2	0.021
Time set (d)				
	EN commenced (dth)	3.6 ± 1.3	6.4 ± 3.7	0.001
	EN up to 60%* (d)	3.8 ± 2.3	1.9 ± 0.8	0.017
PN fluid and ingredients (/d)				
	Total liquid (ml)	2546.2 ± 692.1	2933.2 ± 870.2	0.002
	Glucose (g)	189.3 ± 25.4	187.1 ± 23.3	0.371
	Amino acid (g)	68.7 ± 6.8	67.6 ± 5.9	0.233
	Fat emulsion (ml)	253.7 ± 8.4	259.6 ± 11.5	0.436
	ω-3 PUFA (g)	9.3 ± 0.57	9.6 ± 0.66	0.284
Length of stay (d)		7.7 ± 2.6	10.8 ± 3.8	0.032
Hospitalized cost (CNY)		8841.9 ± 2018.6	10860.6 ± 2803.6	0.014

PPN, peripheral parenteral nutrition. Central venous catheterization PICC, peripherally inserted central catheter, subclavian vein, internal jugular, and femoral vein; TIVAP, totally implantable venous access ports; ONS, oral nutritional supplements; Tube feeding includes nasogastric tube, nasoduodenal tube, and nasal jejunal tubes; PEG, percutaneous endoscopic gastrostomy tube; PEJ, percutaneous endoscopic jejunostomy; PUFA, polyunsaturated fatty acid.

*EN up to 60%, the elapsed time since EN commenced.

Concurrently, the early initiation of EN must be managed to avoid refeeding syndrome. This condition involves a series of metabolic disturbances that occur when nutritional support is reintroduced to malnourished patients, primarily characterized by severe electrolyte imbalances, specifically hypophosphatemia. Abnormal glucose metabolism, sodium balance disorder in the fluid and blood, and fatal arrhythmia are usually accompanied by hypokalemia and low magnesium levels (47, 48). For elderly patients at risk of refeeding syndrome, nutritional support should start with low-energy nutrition and slowly increase to the target demand, and the balance of liquid circulation volume should be strictly monitored. Simultaneously, attention should be paid to supplementing vitamins, trace elements, and electrolytes. When the symptoms of refeeding syndrome recur, we should reduce or even stop energy intake, correct electrolyte disorder, supplement vitamins, and maintain organ function. Patients with incomplete intestinal obstruction caused by short-term fasting of water and loss of digestive tract function need a buffer time to restore gastrointestinal function and can be given 25% of the total target demand at first. Patients with good tolerance can gradually reach the target demand within 3 days; patients with poor tolerance can gradually reach the target demand within 7 days. At the same time, doctors should closely monitor changes in electrolytes (49, 50).

As the body ages, there are many physiological changes to metabolism and body function as well as to body composition and organ function, which affects energy demand, nutrition, and body fluids. Simultaneously, the elderly are more likely to experience diabetes, high blood pressure, coronary heart disease, COPD, and chronic renal failure, as well as organ dysfunction, an insufficient physiological reserve, and a poor reaction to stress. Chewing function is reduced in elderly people, their oral intake is low, their digestion and absorption ability is weak, and they are prone to malnutrition, which has a negative effect on clinical prognosis (51). This negative effect affects tissues and organs, lowering the QOL and increasing the rate of hospital admission and readmission, hospitalization time, complications, and mortality. It also brings difficulties to the diagnosis and treatment of acute and chronic diseases (52).

Based on the physiological characteristics of elderly patients, appropriate screening and assessment tools were used to identify malnutrition and nutritional risk. Under the guidance of an NST, targeted nutritional support was formulated to meet energy and protein targets while controlling total fluid intake to reduce the circulatory burden on the heart, lungs, liver, and kidneys (Table 4).

**Table 4:** Comparison before and after treatment for seven days.

Laboratory indexes	ECPEN group			DCPEN group			Reference values
	BEF	AFT	*P* value	BEF	AFT	*P* value	
WBC	13.29 ± 5.73	7.66 ± 2.28	0.002	14.43 ± 6.82	8.31 ± 3.55	0.013	3.5–10 10^9
Lymphocyte	0.882 ± 0.114	0.542 ± 0.191	0.012	0.851 ± 0.136	0.632 ± 0.123	0.033	0.5–0.7
CRP	7.45 ± 3.83	1.72 ± 0.84	0.351	8.25 ± 5.72	2.29 ± 1.82	0.386	0–0.8 mg/dL
IL-6	149.81 ± 54.22	8.67 ± 3.15	0.001	185.75 ± 69.16	22.05 ± 8.91	0.001	0–5.9 pg./mL
PCT	5.95 ± 3.86	0.89 ± 0.41	0.004	5.67 ± 4.93	0.97 ± 0.36	0.011	<0.5 ng/mL
ALB	30.23 ± 11.22	35.06 ± 10.21	0.002	29.24 ± 13.41	38.15 ± 10.04	0.004	35-50 g/L
PH	7.32 ± 0.13	7.38 ± 0.08	0.341	7.34 ± 0.15	7.39 ± 0.11	0.434	7.35–7.45
Blood glucose	7.75 ± 3.99	6.44 ± 3.12	0.045	8.47 ± 5.18	7.71 ± 4.95	0.604	3.4–6.1 mmol/ L
TC	4.67 ± 1.41	4.25 ± 1.19	0.481	4.89 ± 1.38	4.35 ± 1.08	0.387	3.1–5.7 mmol/ L
Triglyceride	1.43 ± 0.49	1.38 ± 0.34	0.588	1.48 ± 0.39	1.37 ± 0.39	0.692	0.4–1.7 mmol/ L
LDL	4.47 ± 0.84	4.36 ± 0.88	0.596	4.76 ± 0.92	4.53 ± 0.95	0.542	0–3.4 mmol/ L
HDL	1.23 ± 0.13	1.34 ± 0.24	0.458	1.24 ± 0.19	1.32 ± 0.27	0.538	1.0–1.6 mmol/ L
Serum sodium	121.15 ± 25.03	141.65 ± 27.1	0.012	126.49 ± 30.23	137.67 ± 24.5	0.031	130–150 mmol/ L
Serum kalium	3.32 ± 0.34	3.98 ± 0.66	0.018	3.41 ± 0.37	3.63 ± 0.58	0.042	3.5–5.5 mmol/ L
Serum phosphate	0.79 ± 0.24	1.27 ± 0.34	0.002	0.84 ± 0.32	1.19 ± 0.26	0.009	0.89–1.6 mmol/ L
Serum magnesium	0.87 ± 0.18	0.97 ± 0.23	0.406	0.79 ± 0.14	0.85 ± 0.16	0.511	0.6–1.4 mmol/ L
ALT	67.13 ± 26.82	31.3 ± 12.41	0.023	70.11 ± 33.22	58.9 ± 13.85	0.348	0-40 U/L
AST	58.87 ± 25.43	16.86 ± 8.43	0.001	59.67 ± 31.35	45.53 ± 20.53	0.237	0-40 U/L

BEF, before; AFT, after; WBC, white blood cell; CRP, C-reactive protein; IL-6, interleukin-6; PCT, procalcitonin; ALB, serum albumin; PH, pondus hydrogenii; TC, total cholesterol; LDL, low-density lipoprotein; HDL, high-density lipoprotein; ALT, alanine aminotransferase; AST, aspertate aminotransferase.

It is recommended that elderly patients with nutritional risk or malnutrition use an indirect heat-measuring method to detect body resting energy expenditure to determine the target energy demand. If we cannot complete the measurement via the above method, then (104.5–125.4 kJ/kg·d) [25–30 kcal (/kg·d)] will be used as the standard energy support for adults. Due to the decline in metabolic function, 70% of the recommended standard energy for adults can be used as the target energy demand for patients over 75 years old. However, they must factor in the patient’s age, activity intensity, stress levels, and liver and kidney function to correct and adjust.

Daily intake of protein is associated with maintaining a high muscle level (53). Studies have shown that high levels of amino acids in the blood can effectively stimulate the synthesis of muscle protein. The target demand for protein for adults is 1.0–1.5 g (/kg·d). If there is no abnormality in renal function, the target requirement can be appropriately adjusted to 1.2–1.5 g (/kg·d) for elderly patients with simple physical activity (54). If accompanied by acute or chronic renal insufficiency, the target demand in protein is limited to 1.0 g (/kg·d) (55).

For elderly patients with cardiac, pulmonary, hepatic, or renal complications, total infusion volume should be limited. When using fat emulsions, the proportion of fat in both enteral and parenteral nutrition should be increased to enhance energy density In addition, the intake of saturated fatty acids should be reduced as much as possible and medium-chain fatty acids, omega-3 fatty acids, and monounsaturated fatty acids should be increased in order to optimize the proportion of fatty acids, meet the rapid and efficient energy demand, and reduce the metabolic burden on the liver, lipid peroxidation, and the risk of cardiovascular and cerebrovascular disease (56). Studies have demonstrated that *ω*-3 also has anti-inflammatory and immunomodulatory functions. The dosage of 0.2 g (/kg·d) in parenteral nutrition can significantly reduce the levels of IL-6, TNF, PCT, and CD4^+^/CD8^+^ ratio, thus reducing the incidence of infection and systemic inflammatory response syndrome in elderly patients. Although we encourage the energy density of total parenteral nutrition solution to limit fluid replenishment, high permeability can also lead to complications (57). Studies show that, when the osmotic pressure of infusion is higher than 850 mOsm/L, the incidence of thrombophlebitis will increase correspondingly after continuous infusion for more than 10 days (58).

### 3.3 Metabolic stability and safety considerations

Elderly patients’ insulin sensitivity is reduced by 43%, and the risk of diabetes is increased by 16% (59), making them prone to glucose intake and utilization issues (60). Excessive glucose in parenteral nutrition support will lead to blood glucose disorders and induce respiratory failure, fatty liver, and liver dysfunction. If the infusion speed of glucose is higher than 4–5 mg (/kg·min) or the energy of glucose is higher than 125.4 kj/(/kg·d), oxidation rate will overload the metabolic capacity, leading to hyperglycemia, fat accumulation, and fatty infiltration in the liver (61). Therefore, the glucose content in parenteral nutrition rehydration should be strictly controlled to increase the fat-to-energy supply ratio, which is beneficial for elderly patients. The results show that the dosage of glucose was limited to 2.5 g (/kg·d), accounting for 50–55% of the total calories in elderly patients with parenteral nutrition, which meets their physiological needs and extra consumption and does not increase the risk of blood glucose imbalance, respiratory failure, or liver and renal insufficiency (62).

Immunoenhancers are standard-based nutritional preparations. By adding glutamine, arginine, omega-3 polyunsaturated fatty acids, nucleotides, and other specific nutritional substances, the metabolism and immune function of the body can be regulated through pharmacological actions. It is very important to maintain lean body weight, promote the repair of intestinal mucosal injury, reduce infection complications, and shorten hospitalization time (63). However, we still need to be cautious in using immune-enhancing materials due to the side effects of immune nutrients. For critically ill patients, especially those with severe septic shock and microcirculatory disturbance, conventional immune enhancement is not recommended (Table 5).

**Table 5:** Clavien-Dindo grade.

	ECPEN group	DCPEN group	*P* value
Complications in 30 days (%)			0.027
No complication	34 (43.0)	23 (29.1)	
I	21 (26.6)	17 (21.5)	
II	10 (12.7)	22 (27.8)	
III	8 (10.1)	7 (8.9)	
IV	6 (7.6)	10 (12.7)	
Complications in the short-term (%)			0.034
No complication	40 (50.6)	33 (41.8)	
I	17 (21.5)	12 (15.2)	
II	15 (19.0)	25 (31.6)	
III	5 (6.3)	5 (6.3)	
IV	2 (2.5)	4 (5.1)	
Readmissions			0.046
Within 30 days	3 (3.8)	5 (6.3)	
Within 90 days	7 (8.9)	13 (16.5)	
Within 180 days	20 (25.3)	31 (39.2)	

Complications in 30 days include intestinal obstruction, intestinal infection, pneumonia, diarrhea, vomiting, thrombosis, pressure sore, edema, weight loss, surgical invention, and death.

Short-term (within 180 days) complications include sarcopenia, pneumonia, thrombosis, pressure sore, weight loss, recurrence of obstruction, surgical invention, and death.

Following hospital discharge, the body often remains in a catabolic state after the stress of an acute illness. The ordinary daily diet cannot fully meet the needs of body metabolism, could cause a gradual decline in body mass, and negatively affect the functions of tissues, cells, and organs. Therefore, we need to continue to use ONS to improve nutritional supplements. Family ONS nutritional support can last for two weeks to several months and can promote physical recovery, reduce the incidence of complications and readmission, and improve QOL (64). Besides the daily diet, ONS should be at least 400–600 kcal/day. We suggest that NST experts participate in re-evaluating and screening the nutritional status of patients and adjust nutritional treatment plans over time.

From this study, we can draw the conclusion that old age is not a contraindication to EN in the early stage of incomplete intestinal obstruction. By estimating the time to apply EN accurately, allocating EN and PN rationally, and arranging the total amount of intravenous fluid based on the patient’s organic function, EN can benefit elderly patients and be an important nutritional support for incomplete ileus through non-operative treatment. Although our research has achieved some results with clinical guiding value, more prospective multi-center controlled studies are needed to verify the correctness of the retrospective research results. One limitation of this study is the relatively small sample size. However, this is due to our focus on a very specific geriatric population (≥80 years) and the implementation of strict inclusion criteria and PSM to ensure data quality and comparability. In the future, more scientific treatment plans will be developed through clinical research, and elderly patients with intestinal obstruction will benefit from thema.

## Data Availability

The original contributions presented in the study are included in the article/supplementary material, further inquiries can be directed to the corresponding author/s.
